# Dynamic changes during the treatment of pancreatic cancer

**DOI:** 10.18632/oncotarget.24483

**Published:** 2018-02-13

**Authors:** Robert A. Wolff, Andrea Wang-Gillam, Hector Alvarez, Hervé Tiriac, Dannielle Engle, Shurong Hou, Abigail F. Groff, Anthony San Lucas, Vincent Bernard, Kelvin Allenson, Jonathan Castillo, Dong Kim, Feven Mulu, Jonathan Huang, Bret Stephens, Ignacio I. Wistuba, Matthew Katz, Gauri Varadhachary, YoungKyu Park, James Hicks, Arul Chinnaiyan, Louis Scampavia, Timothy Spicer, Chiara Gerhardinger, Anirban Maitra, David Tuveson, John Rinn, Gregory Lizee, Cassian Yee, Arnold J. Levine

**Affiliations:** ^1^ Department of Gastrointestinal (GI) Medical Oncology, MD Anderson Cancer Center, Houston, TX, USA; ^2^ Division of Oncology, Washington University, St. Louis, MO, USA; ^3^ Cold Spring Harbor Laboratory, New York, NY, USA; ^4^ Department of Molecular Therapeutics, The Scripps Research Institute, Jupiter, FL, USA; ^5^ Center for Translational Pathology, University of Michigan Medical Center, Ann Arbor, MI, USA; ^6^ Department of Molecular and Cellular Biology, Harvard University, The Broad Institute, Cambridge, MA, USA; ^7^ Simons Center for Systems Biology, Institute for Advanced Study, Princeton, NJ, USA; ^8^ Department of Pathology, MD Anderson Cancer Center, Houston, TX, USA; ^9^ Department of Translational Molecular Pathology, MD Anderson Cancer Center, Houston, TX, USA; ^10^ Department of Radiation Oncology, MD Anderson Cancer Center, Houston, TX, USA; ^11^ Department of Surgical Oncology, MD Anderson Cancer Center, Houston, TX, USA; ^12^ Department of Melanoma Medical Oncology, MD Anderson Cancer Center, Houston, TX, USA; ^13^ Current address: University of Colorado Boulder, BioFrontiers Institute, Boulder, CO, USA

**Keywords:** pancreatic cancer, genomic instability, organoids, epithelial-mesenchymal transition

## Abstract

This manuscript follows a single patient with pancreatic adenocarcinoma for a five year period, detailing the clinical record, pathology, the dynamic evolution of molecular and cellular alterations as well as the responses to treatments with chemotherapies, targeted therapies and immunotherapies. DNA and RNA samples from biopsies and blood identified a dynamic set of changes in allelic imbalances and copy number variations in response to therapies. Organoid cultures established from biopsies over time were employed for extensive drug testing to determine if this approach was feasible for treatments. When an unusual drug response was detected, an extensive RNA sequencing analysis was employed to establish novel mechanisms of action of this drug. Organoid cell cultures were employed to identify possible antigens associated with the tumor and the patient’s T-cells were expanded against one of these antigens. Similar and identical T-cell receptor sequences were observed in the initial biopsy and the expanded T-cell population. Immunotherapy treatment failed to shrink the tumor, which had undergone an epithelial to mesenchymal transition prior to therapy. A warm autopsy of the metastatic lung tumor permitted an extensive analysis of tumor heterogeneity over five years of treatment and surgery. This detailed analysis of the clinical descriptions, imaging, pathology, molecular and cellular evolution of the tumors, treatments, and responses to chemotherapy, targeted therapies, and immunotherapies, as well as attempts at the development of personalized medical treatments for a single patient should provide a valuable guide to future directions in cancer treatment.

## INTRODUCTION

On December 22, 2011, a patient was diagnosed with an inoperable pancreatic adenocarcinoma. After Folfirinox therapy, the tumor regressed and became operable. It was removed on May 30, 2012. Eighteen months later, metastatic lesions appeared in the lungs and on the rib cage. It was at this point (Nov.1, 2013) that the family decided to assemble a group of scientists who had interest in and could carry out extensive surveillance of the tumor in the patient and perform experimentation with clinical materials collected from blood and biopsies of the tumor tissue. The purpose of this was to inform and assist the clinicians who were making the decisions about the treatment and care of their patient.

**Figure 1:**
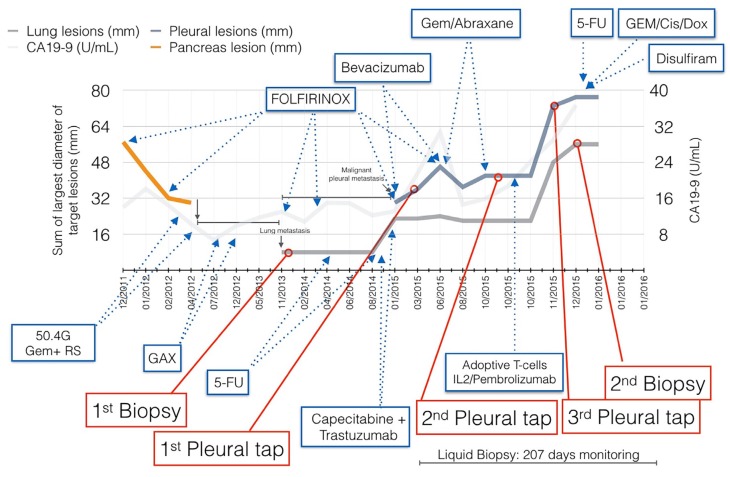
Patient clinical course throughout a four year period Size of lung and pleural metastatic lesions were used to determine response to therapies (left axis). CA-19-9 levels are plotted, although remain mostly within normal range (right axis).

The goals of this project were broader than the care of a single patient: to follow a number of different parameters during the dynamic evolution of a tumor in a patient. This was accomplished by obtaining serial biopsies and cancer cells and DNA from blood samples over the entire time of treatment. A time line was created, employing DNA sequencing from blood samples, the production of three dimensional, or organoid, tissue cultures and two-dimensional cell cultures, from serial biopsies (as well as DNA and RNA sequencing of these tissues) in order to follow the changes occurring during treatments, and tumor response and progression (Figure 1). Tumor heterogeneity was explored, detected, and followed over time. Possible tumor antigens were identified from biopsy samples and organoids. T-cell receptor sequences were determined from the peripheral blood and the T-cells located in tumor tissue. T-cells from the peripheral blood of the patient were expanded with antigen exposure for possible therapeutic choices. Attempts at making CAR-T-cells directed against an antigen observed on the tumor and tested in the organoid cultures were explored. Organoid cultures were employed to test for drug sensitivities in cell culture and in mice and the results were compared with expected sensitivities based upon DNA mutational profiles observed in the tumors. When some unexpected drug sensitivities were identified, an extensive RNA sequencing analysis was carried out with tumor tissue from mice treated with these drugs and possible pathways involved in this treatment were elucidated.

Many of the experimental approaches described here were carried out for the first time in cell culture using biological materials directly or recently from a patient. As such, they represent the forerunner of future paths to be taken for the benefit of many patients. In addition, these experiments addressed a limitation of past DNA sequencing studies with tumor tissue, which explore only one time point in the evolution of a tumor. This dynamical study follows the changes in DNA sequences and DNA sequence heterogeneity with time and treatment (albeit for only a single patient). The DNA sequences obtained from samples in the blood were compared to those sequence changes found in the organoids made from biopsies taken at different times. One of the important lessons learned from these studies is that the DNA sequences, epigenetic states, and properties of cancers evolve with time and changing treatment protocols. Precision medicine aspires to act upon the biological information about a tumor, to craft an informed and intelligent treatment response. We will have to learn how to obtain information about the tumor rapidly, so as to intervene before the tumor changes its basic information. This report reflects an attempt to do just that.

The authors of this unusual research venture formed a team to exchange biological samples, information, new experimental protocols, and novel ideas and directions. They often held joint meetings and phone conferences to stay focused, organized, and informed about new observations. They all sacrificed time and effort from other research projects and activities, because they all came to appreciate the rewards of helping an individual patient, perhaps more directly than previous research avenues in a laboratory had permitted.

## CLINICAL BACKGROUND

A previously healthy, 50-year-old Jewish man presented with intermittent back pain and epigastric discomfort beginning in 8/2011. Computed tomography of the abdomen in December 2011 revealed a 7.3 cm x 2.9 cm mass in the body of the pancreas, encasing the splenic artery and extending along the celiac axis. The mass was also abutting the proximal common hepatic artery and superior mesentery artery. Endoscopic ultrasound with fine needle aspirate of the pancreatic mass was performed with cytology positive for adenocarcinoma. The patient’s family medical history was notable for a sister with breast cancer in her 60s and a father with prostate cancer. Carbohydrate antigen 19-9 was within normal limits and germline mutational testing was negative for a deleterious BRCA 1 or 2 mutation. The majority of the patient’s clinical care was coordinated between MD Anderson in Houston and Siteman Cancer Center in St. Louis.

In light of the tumor’s extensive vascular involvement, the patient was started on 5-FU, leucovorin, oxaliplatin and irinotecan, FOLFIRINOX). After a total of 4 cycles of chemotherapy, there was radiographic evidence of response. The patient received 1 more dose and he then proceeded with chemoradiation. The patient received gemcitabine-based chemoradiation to a total radiation dose of 50.4 Gy with one dose of bevacizumab at the initiation of radiotherapy and 2 weekly doses of cetuximab as radiation completed. The patient tolerated the treatment well, and follow-up CT imaging revealed significant tumor regression. Tumor shrinkage was seen on repeat imaging.

The patient was referred to the Medical College of Wisconsin and underwent an R0 distal pancreatectomy and splenectomy; the common hepatic artery was spared. Pathology confirmed a stage IIA (T3N0M0) pancreatic cancer with significant treatment-effects response. The patient’s post-operative course was uneventful, followed by two months of adjuvant therapy comprised of gemcitabine, capecitabine and nab-paclitaxel.

Close radiographic and clinical surveillance for relapse revealed no evidence of disease until November 2013, 18 months postop. At that time, small lung lesions and a rib lesion were noted. The patient and his family assembled a team of clinicians and scientists that could provide further expert clinical guidance and devise scientifically sound novel therapeutics. Thorascopic wedge resection of a left lung lesion confirmed metastatic disease with residual tissue used to create organoids and patient derived murine xenografts. The patient resumed FOLFIRINOX treatment along with denosumab. The patient had a minor radiographic response to therapy and after 4 months of therapy, he was placed on maintenance infusional 5FU with disease remaining stable through November 2014. During this interval, single cell analysis from tumor derived organoids demonstrated amplification of HER2 and the patient was tried on capecitabine plus trastuzumab. Tumor progression was documented after a 6-week course of therapy.

In February 2015, shortly after a brief chemotherapy holiday, the patient presented with shortness of breath caused by a new onset of right pleural effusion. Malignant cells were seen in the pleural fluid. FOLFIRINIX was resumed with the addition of bevacizumab. Bevacizumab was discontinued after asymptomatic pulmonary embolism was noted. Anticoagulant therapy was initiated and FOLFIRINOX was continued. By June 2015, the patient had disease progression in the lung, and he was started on gemcitabine and nab-paclitaxel with paricalcitol. The patient had a minor response to gemcitabine, nab-paclitaxel, and paracaltriol for 2 months with progression after 2 more months of treatment.

In October 2015, the patient enrolled in a clinical trial at MD Anderson, which administered autologous T-cells enriched for reactivity to VMMR-1. A non-mutated antigen was found, based on organoid peptide analysis. Based on the study protocol, he received Cytoxan, followed by T cell infusion, then subcutaneous IL-2 injections and pembrolizumab. The patient experienced significant weight loss and fatigue during the therapy. He required frequent therapeutic thoracenteses for palliation of dyspnea. A repeat CT scan of the chest in 11/2015 showed rapid disease progression, manifested as an interval increase in lung lesions and pleural-based metastatic disease. Surprisingly, the biopsy of a pleural-based nodule revealed a poorly differentiated neuroendocrine tumor.

In light of the rapid disease progression, a combination of 5-FU, gemcitabine, docetaxel and cisplatin was initiated on 12/14/2015. The patient’s respiratory status deteriorated and he was intubated two days later. Concern of an acute inflammatory response was raised, and high dose steroid therapy was instituted. A similar chemotherapy regimen was administered two weeks later, and the repeat CT revealed stable pleural caking and lung metastatic lesions. Although tumor stabilization was achieved with resumption of cytotoxic chemotherapy, he remained with restrictive lung disease and he opted for a tracheostomy for persistent hypoxemia and hypercarbic respiratory failure requiring mechanical ventilation. He had clear mentation with intact organ functions.

On January 6, 2016, the patient was transferred to Barnes Jewish Hospital in St. Louis. He was treated with carboplatin and etoposide for poorly differentiated neuroendocrine tumor histology. Therapy was delivered in an intensive care unit, and he subsequently transferred to an immediate care floor for ventilator care given his stable clinical condition. A repeat FNA of the pleural lesion was performed and cytology revealed mixed histology malignancy with predominant neuroendocrine tumor in addition to features of adenocarcinoma. Meanwhile, the patient had suffered a ventilator-related tracheostomy infection, pneumonia, fungal infection and bilateral lower extremities DVT. His clinical condition declined over the hospital stay despite normal neurocognitive function and adequate cardiac, renal, and hepatic function. The patient requested ventilator support be withdrawn and he expired shortly thereafter, 50 months from his initial diagnosis of pancreatic cancer.

### Evolving genomic landscapes of a metastatic PDAC patient through liquid biopsies and multi-region sequencing

**Figure 2:**
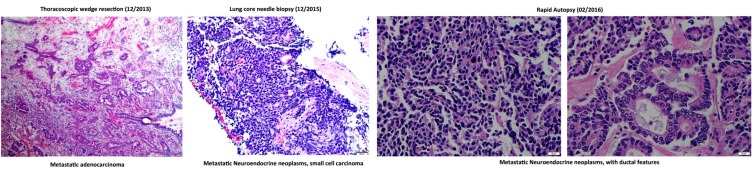
H&E staining of lung metastatic biopsy taken at the beginning (left) and end (middle and right) of liquid biopsy follow up Histological analysis demonstrates a phenotypic change from adenocarcinoma to small cell-like tumor. Rapid autopsy samples show phenotypically distinct lesions comprising neuroendocrine and ductal-like histologies.

Fifteen months after the wedge resection, the patient began to be monitored by the liquid biopsy program at MD Anderson in the Maitra lab. A total of eighteen liquid biopsies (15 peripheral blood and 3 malignant pleural effusions) were performed throughout disease progression and therapeutic intervention, which include chemotherapies, targeted therapies, and immunotherapies. Subsequently, the patient underwent a second lung biopsy (a core needle biopsy). The core needle biopsy (CNB) was histologically determined to be a metastatic neoplasm with high-grade neuroendocrine features (Figure 2). At the time of death, rapid autopsy was performed on the patient, at which point seven spatially distinct lung metastatic lesions acquired for molecular analysis. Disease response was primarily determined by CT imaging as the standard clinical biomarker, CA-19-9, remained within normal ranges throughout disease course. The tissue and liquid biopsies were utilized as an ancillary decision support for this patient, as follows:

### *KRAS* and *TP53* mutations as surrogates of patient tumor burden

Next-generation sequencing (NGS) analysis of both the patient’s initial metastatic tumor (histologically consistent with an adenocarcinoma) and second lung metastasis (histologically consistent as a high grade neuroendocrine carcinoma) samples harbored oncogenic *KRAS* G12D and *TP53* R175H mutations, along with several truncal genomic imbalances, with concordant changes in gene expression (*ERBB2*, *FOXO1*, *KLK2*, *NRAS*, *DAXX*, *HMGA1* and *NOTCH2* amplifications and a *FHIT* deletion) suggesting that the metastatic small cell carcinoma was not a new primary tumor, but rather an evolution of the original metastatic PDAC.

**Figure 3:**
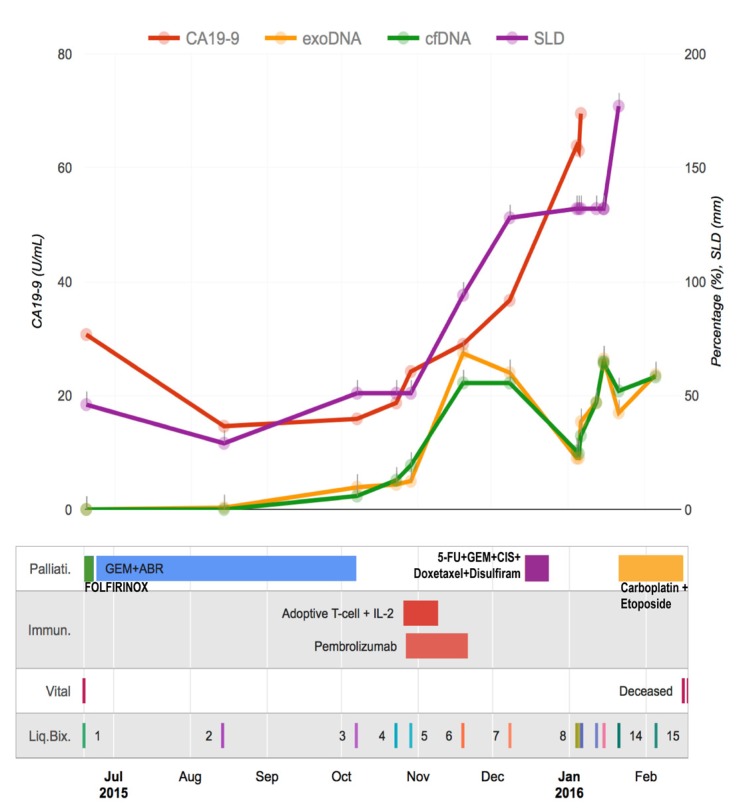
Treatment monitoring for a metastatic PDAC patient with 15 liquid biopsies The graph illustrates the rise and fall of molecular and clinical indicators in the context of cancer treatments.

Since *KRAS* represents a reliable tumor burden surrogate for PDAC (seen in close to 95% of PDAC) it was decided to perform ultrasensitive droplet digital PCR (ddPCR) in the complete series of 15 blood based liquid biopsies [1]. Mutation allele frequencies (MAFs) of the driver *KRAS* in both the exosomes-derived DNA (exoDNA) and circulating cell-free DNA (cfDNA) were plotted against clinical covariates (Figure 3). The MAFs for both compartments trended with patient tumor burden, including size of the pulmonary metastases (sum of the largest dimension, “SLD” according to RECIST 1.1 criteria), suggesting that these mutations are representative of tumor burden. Notably, the tumor burden (as determined by exoDNA and cfDNA mutant *KRAS* allele fraction) rose from non-detectable to detectable levels when therapy was changed from FOLFIRINOX to Gemcitabine/Abraxane, and thereafter, sharply spiked after the patient received adoptive T-cell therapy, finally regressing once the patient resumed cytotoxic chemotherapy comprised of a “cocktail” of agents, including cisplatin, carboplatin and etoposide (amongst others).

### Landscapes of genomic heterogeneity in metastatic tumor tissue

**Figure 4:**
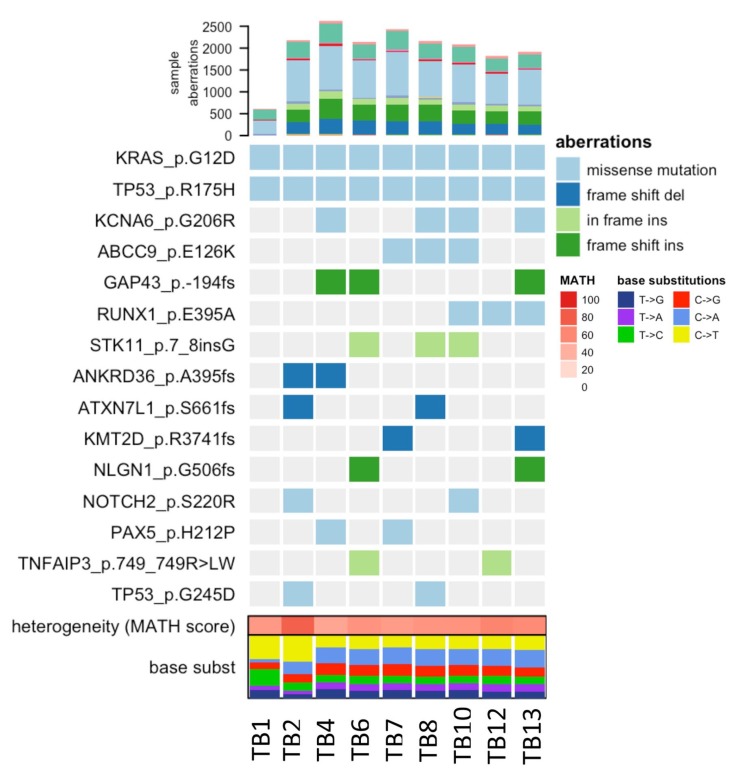
Heatmap of COSMIC mutations detected across tissue samples Only those mutations present in at least two distinct lesions were considered based on a consensus approach. TB1 and TB2 corresponds to a wedge and core biopsy acquired on 12/2013 and 12/2015 respectively. TB4-TB13 are tissues acquired from rapid autopsy on 02/2016.

At the time of death, rapid autopsy retrieved seven warm biopsies of the lung tissue metastasis, representing distinct spatial lesions, with the goal of determining patterns of heterogeneity. Tumor regions were separated by a margin of at least 0.5cm in order to be considered a spatially distinct lesion, and were compared to the two prior biopsy samples (Figure 4 TB1 and TB2). Significant heterogeneity among putative subclonal driver mutations began to accumulate over time, and these were partially shared across tissue samples. These included mutations in *STK11* and *NOTCH2*, which have been previously described to have a role in PDAC carcinogenesis, and a secondary mutation in *TP53,* which may have been related to selective pressures induced by this patient’s prolonged therapeutic history [2, 3].

### Allelic imbalances

**Figure 5:**
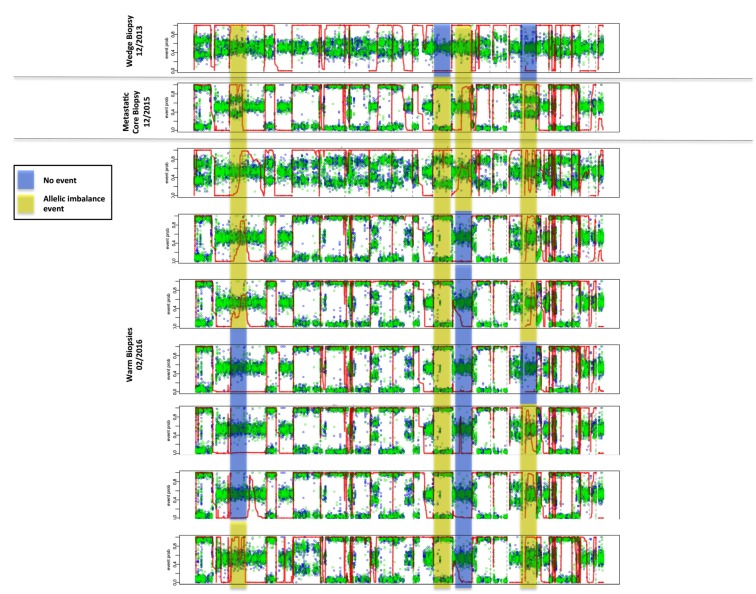
Detection of allelic imbalance events by *hapLOHseq* across sequenced tissue samples Representative events are highlighted to depict distinct patterns of chromosomal aberrations across tissue sites.

Identification of chromosomal allelic imbalance (AI) may indicate ongoing chromosomal instability, and can be inferred through whole exome sequencing. As a result of chromosomal deletions or duplications, AI profiles provide insights into patterns of tumor progression, with potential correlates to prognosis and therapeutic actionability. In order to overcome the challenges of detecting subtle chromosomal AI, due to diluted tumor fractions such as those seen through liquid biopsies, the MD Anderson team applied a methodology known as hapLOHseq [4]. In doing so, they sought to identify unique temporal and spatial patterns of AI in distinct biopsies taken throughout a more than two-year period. As depicted in Figure 5, they observed pre-existing AI events on chromosomes 2 and 11, present since the wedge biopsy (genomic regions shaded in yellow) taken from 2013, but spatially diverse in subsequent multiregion whole exome sequencing two years later. Further events on chromosomes 10 and 15 (shaded in blue) demonstrate the emergence of aberrations that may be directly related to the evolution of this tumor genome.

### Copy number aberrations

**Figure 6:**
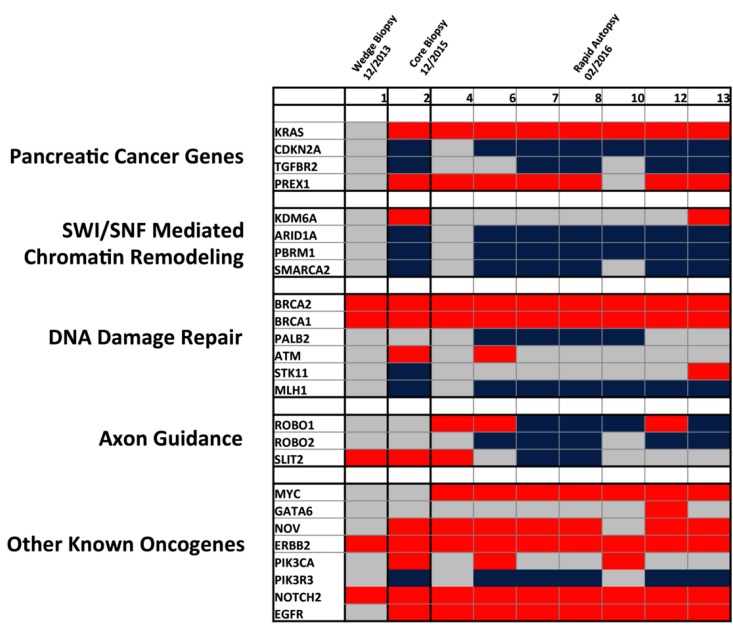
Heatmap of detected copy number variations detected across tissue samples Only CNVs previously associated to PDAC related core pathways are represented based on previous ICGC and TCGA data. Red = amplification, Blue = deletion, and Grey = no CNV.

Although progression of PDAC has been suggested to occur over a period of nearly two decades, affording a window of promise for early detection biomarkers, recent studies have suggested that rather than a step-wise molecular sequence of aberrations, PDAC tumorigenesis may follow a model similar to what is termed punctuated equilibrium [5]. Specifically, it is possible that most mutations accrue in an extended phase under pre-neoplastic tumor development, but a single transformative event, which may be influenced by genomic instability from copy number variations (CNVs), induces a transformative effect facilitating overt metastasis. This underlies the functional importance of CNVs in the PDAC tumorigenesis and potential mechanisms of therapeutic resistance. Thus, patterns of copy number evolution were studied over time to determine if these events were related to disease status. As depicted in Figure 6, significant heterogeneity of copy number changes is present among previously identified PDAC related core signaling pathways [1, 6-8]. CNVs present in the initial wedge biopsy appear to be rare among PDAC related core pathways, although it is important to note the identified amplification in *ERBB2,* which led to targeted therapy selection using Trastuzumab, to which the patient did not respond. Subsequent tissue profiling through a core biopsy showed that the patient’s tumor harbored amplification and overexpression in the *EGFR* gene, potentially providing a resistance mechanism to this therapy.

With tumor progression, clonal aberrations appeared among *KRAS* and *CDKN2A,* as well as genes related to SWI/SNF mediated chromatin remodeling. Of particular interest, is the emergence of *MYC* amplifications detected in all rapid autopsy lesions. Although this was not found in the core biopsy two months prior, it is possible that this CNV was missed due to limited sampling of spatial heterogeneity from a single core biopsy. A greater degree of heterogeneity appears to be present among axon guidance and other oncogenic pathways, which include amplifications and deletions of the same gene across spatially distinct tissue samples (ROBO1 and GATA6). Spatial heterogeneity among tissue samples also exists in genes such as *PIK3CA*. Of note is a significant amplification of DNA damage repair genes (*BRCA1* and *BRCA2*), which may be related to compensatory mechanisms of DNA repair in an unstable genomic background. It is important to note, however, that distinguishing evolutionary copy number events from stochastic aneuploidy may be challenging, underlining the importance of observing such events in a relevant context.

### Inferring mutational signatures in PDAC

**Figure 7:**
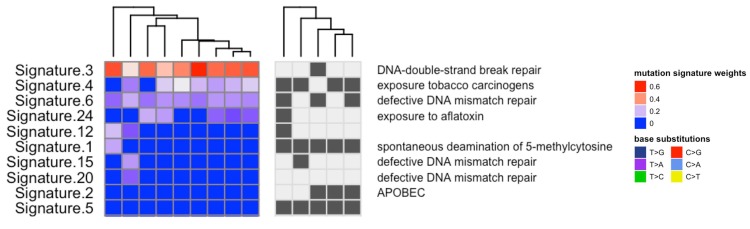
Mutation patterns were defined for each tumor sample using inferred exome sequencing mutations These signatures were then decomposed into putative combinations of known mutation signatures (using COSMIC mutation signatures), where signatures 3, 4 and 6 were highly prevalent across the tissue samples and have previously been associated with deficiencies in *DNA-double-strand break repair*, *exposure to tobacco carcinogens* and *defective DNA mismatch repair* respectively.

Elucidation of mutational signatures across tissue samples revealed the presence of previously described “BRCAness” mutational patterns, which are representative of unstable genomes (Figure 7) (1). Signatures that likely are a consequence of DNA repair deficiency include COSMIC mutation signature 3, which is associated with deficiencies in *DNA-double-strand break repair,* and signature 6, which is associated with defective *DNA mismatch repair*. This BRCAness signature is putatively associated with DNA Damage Repair (DDR) aberrations in cancers, for which DNA damaging drugs may prove effective, such as platinum compounds like cisplatin and oxaliplatin, or PARP inhibitors. Tumor tissue profiles experienced an increase in load of genomic aberrations (specifically, structural variations) over time, suggestiing that the patient’s tumor was, in fact, DDR deficient. Correlated to this hypothesis, the patient consistently responded to therapies involving platinum based chemotherapies such as FOLFIRINOX and readily progressed when switched to Gemcitabine based regimens.

### PARP overexpression underscores a state of ongoing DNA repair defect

**Figure 8:**
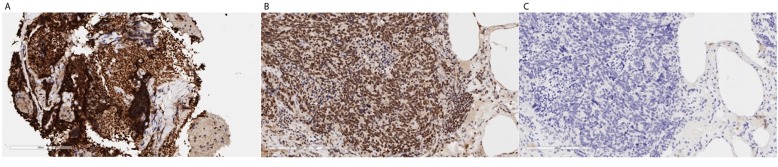
**A.** The biopsy is a CNB with a lot of crush artifact, but PARP IHC seems strongly positive as compared with a known positive control of a human SCLC. **B.** (Thermo cat# RB-1516-P, rabbit polyclonal, 1:100). Panel **C.** represents a negative control without primary antibody.

To further elucidate whether alternative DNA repair mechanisms were active in the tumor, RNA sequencing analysis was performed on the last core biopsy, demonstrating high expression of PARP (128.62 TPM) in the tumor (6 fold more expressed than normal pancreas in TCGA). Furthermore, immunohistochemical analysis confirmed PARP overexpression within the high-grade neuroendocrine tumor compartment (Figure 8). Overexpression of this protein is seen in a variety of cancers, and can be associated with poorer overall survival. Moreover, PARP overexpression, in the context of cancer harboring mutations in DDR pathway, leads to the option of using PARP inhibitors as a potential therapeutic strategy. Further evidence supporting this hypothesis comes from the noticeable, albeit transient, reduction in tumor burden, upon administration of a cisplatin-containing regimen (Figure 8).

The MD Anderson studies show the potential clinical utility of liquid biopsies for the monitoring and clinical guidance of an advanced PDAC patient. They demonstrate that circulating MAFs of driver mutations can be representative of tumor burden and that these values correlate with clinical response. These molecular measures are shown to be especially useful in the case of the subject patient, where CA19-9 (a traditional tumor burden measure) was within normal ranges during the majority of his care. The studies demonstrate the temporal and spatial genomic landscapes of this patient’s tumor, identifying arising putative driver aberrations in the form of single nucleotide variants, copy number changes, and gross chromosomal imbalances, depicting the inherent heterogeneity that is present within PDAC. Based on these molecular signatures, it is subsequently possible to select for candidate therapies that may be most beneficial to the patient.

### Organoids, cell lines, sequencing and therapeutics

**Figure 9:**
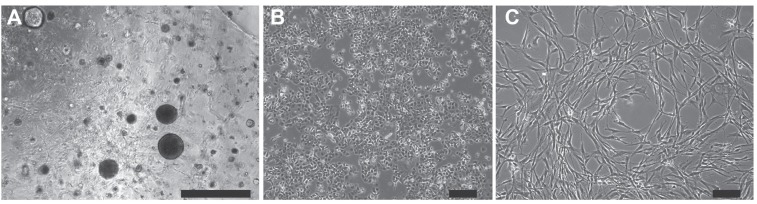
Isolation of hM1 cultures from a lung metastasis hM1 cancer cells were isolated and grown as a three-dimensional organoid **A.**, and adapted to monolayer culture **B**. Cancer associated fibroblast (hM1-CAF) were isolated as a monolayer culture **C**. (Scale bars = 200 µm).

Following the surgical resection of the lung metastasis and the confirmation of pancreatic adenocarcinoma by pathologists at MD Anderson Cancer Center, the Tuveson lab at Cold Spring Harbor Laboratory received four different biopsy specimens, each consisting of multiple tissue fragments. Each sample contained differing contributions of tumor and normal cells and was processed independently as a different sub-line of human metastatic organoid 1 (hM1) (labeled A - D). These samples were used to prepare organoids, and a small portion was formalin fixed and paraffin embedded for independent validation of neoplastic content and cellular composition. Within 48 hours, small organoids were detectable in each individual isolate. Upon passaging, the Cold Spring Harbor Laboratory team began evaluating modifications of the basal media using parameters that were being established for human normal and tumor pancreatic organoid lines [9]. This optimization allowed exponential expansion of the hM1 organoids (Figure 9).

**Figure 10:**
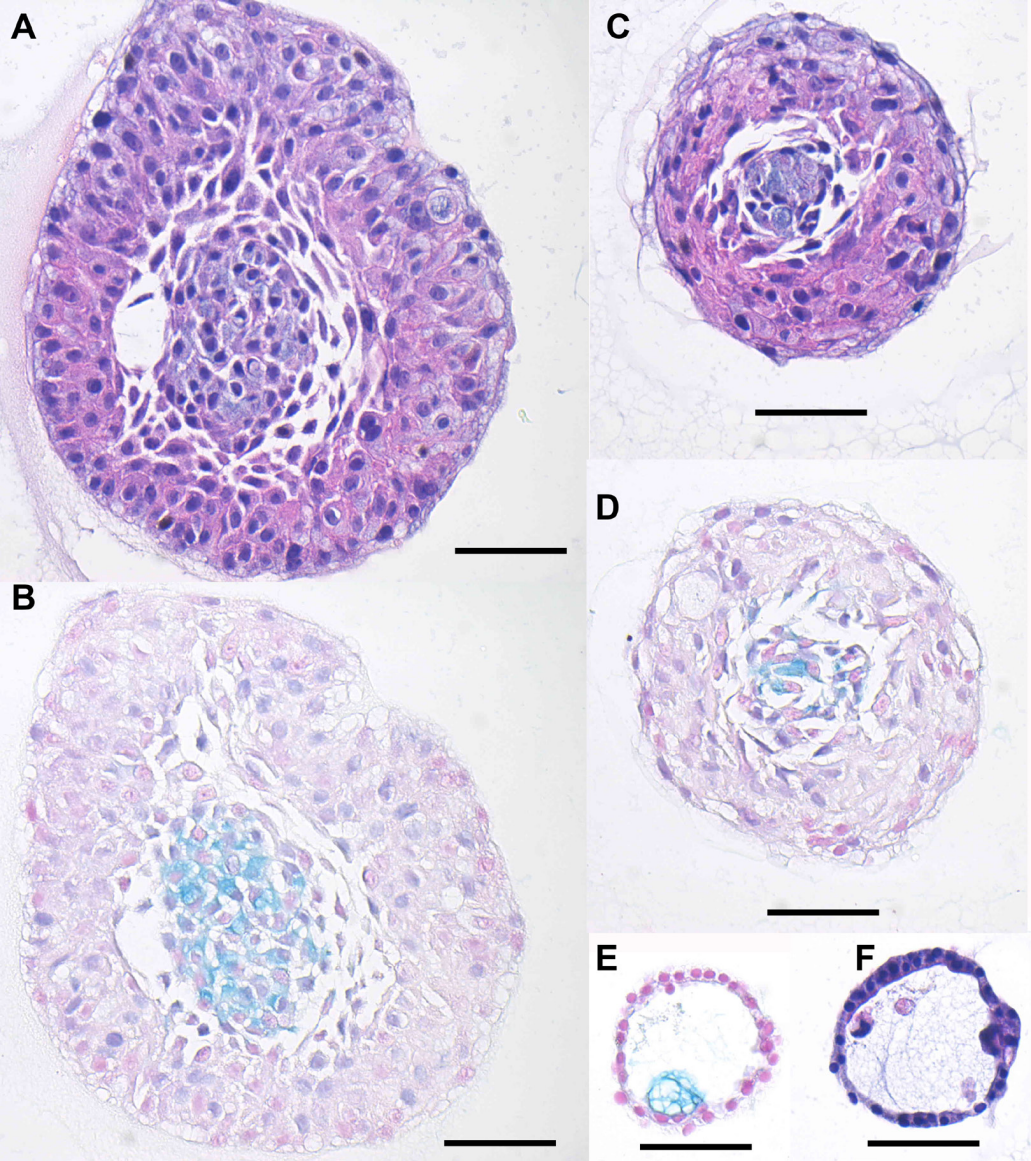
Histologic analysis of hM1 organoids Representative images of the different morphologies by H&E **A.**, **C.**, **F.** and Alcian Blue/Mucin-production stain **B.**, **D.**, **E.** (Scale Bar = 50µm).

In parallel to organoid isolation, a cancer-associated fibroblast line (hM1-CAF) and an organoid-derived 2D cancer cell line (hM1-2D) were established. Generation of a monolayer cell line directly from the patient specimen failed. In addition, patient derived xenografts were established from four fragments from which one tumor engrafted and was passageable following six months of growth *in vivo*. The morphology of the organoids consisted of both single- and multi-layer spheroids (Figure 10), similar to previously generated human organoid lines. Although the patient did not exhibit serological elevation of the pancreatic cancer biomarker, CA19-9, it was highly expressed in the organoids, using flow cytometry and immunoblot (data not shown). The findings suggest that this biomarker is expressed by the tumor cells, but not shed in large amounts, as observed in some PDAC patients.

**Figure 11:**
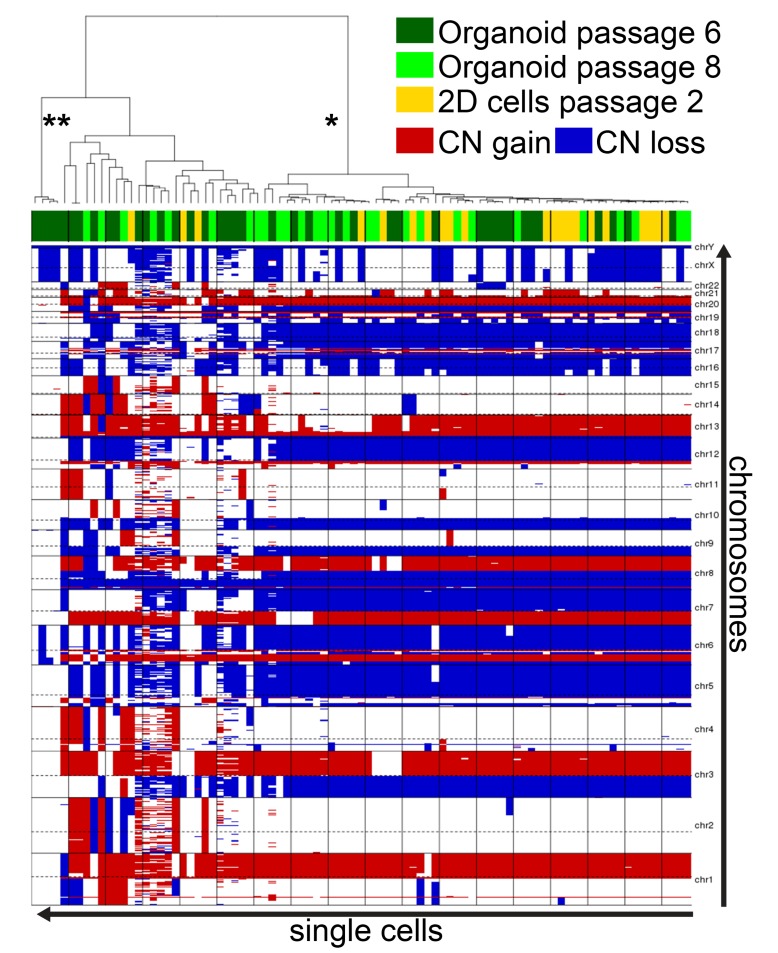
hM1 DNA copy number analysis heatmap Single cells derived from organoids at passage 6 and 8, and from monolayer culture at passage 2 were analyzed for DNA copy number (CN) gain or loss. The predominant clonal features appear in all three cultures (*), while normal-like cells are only present in early passages of the organoid cultures (**).

**Figure 12:**
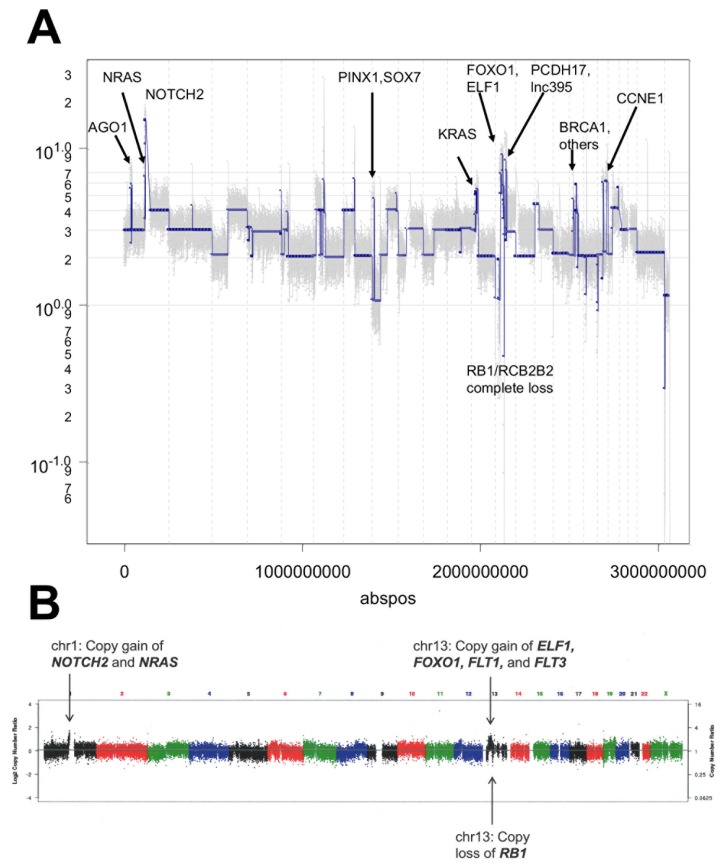
Copy number analysis of hM1 **A.** Copy number analysis of a representative single cell clone of hM1. **B.** Copy number of primary metastatic tissue after laser dissection (∼40% neoplastic cellularity).

To better assess the heterogeneity of the hM1 organoid culture and to discern potential therapeutic avenues, copy number analysis (CNA) was at the single cell level. To account for potential genetic selection in the cultures, analysis was performed on hM1A organoids at two different passages, as well as cells derived from a monolayer culture of hM1A that were established using early passage organoids. This experiment tested 48 single cells derived from two different passages of the organoid culture and compared them to 48 cells grown as a monolayer. DAPI-stained DNA from single nuclei was amplified and analyzed using next generation sequencing to determine copy number variation (Figures 11 and 12A). This analysis revealed that hM1A organoid cultures are oligoclonal and display distinct gene copy number gains and losses, which are recapitulated both in 2D and 3D cultures. The single cell CNA of organoids mirrors the genetic analysis performed at the University of Michigan using frozen metastatic tissue and also reveals distinct and numerous copy number alterations that are not apparent in bulk tumor analysis. (Figure 12A and 12B).The method provides a highly detailed genomic landscape that illustrates which molecular mechanisms are altered in the tumor. For instance, the complete loss of RB and the amplification of Cyclin E1 would lead to a loss of the G1/S cell cycle checkpoint. In addition, the Cold Spring Harbor Laboratory Team study predicts a strong activation of the Mitogen-Activated Protein Kinase pathways, given that KRAS, EGFR, and HER2 are amplified (4.95, 3.79, and 3.05 copies per cell on average, respectively).

**Figure 13:**
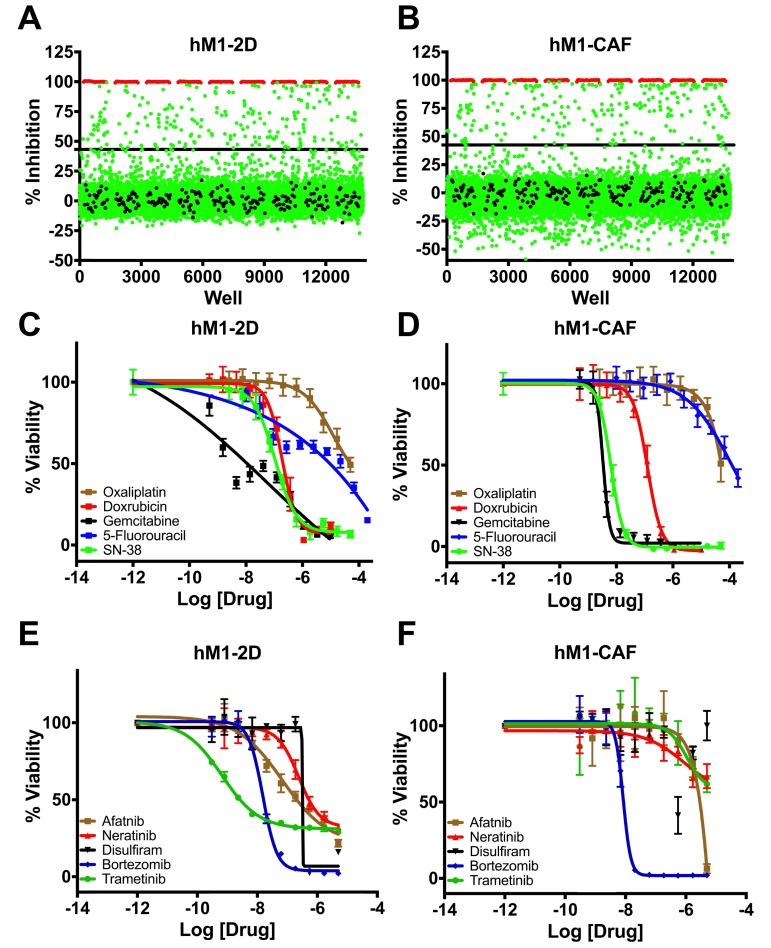
High throughput therapeutic screen of hM1 cells identifies high efficacy targeted inhibitors Growth inhibition effect in hM1 cancer cell line **A.** and in cancer associated fibroblasts **B.** in response to 3200 approved agents. Dose response curves for cytotoxic chemotherapeutics **C.** and **D.** and selected targeted inhibitors in therapeutic screen **E.** and **F.**

The ultimate goal of the Cold Spring Harbor Lab team was to identify and exploit potential therapeutic strategies against hM1 organoids. The single cell CNA highlighted CDK and the ErbB pathway as potential therapeutic targets. The CSHL group also initiated collaboration with the Scripps Research Institute High Throughput Screening (HTS) core to empirically discover additional therapeutic approaches. The goal of the collaboration was to perform unbiased screening of FDA- approved compounds that could be rapidly applied in the clinic. The Scripps Institute compiled a library of ∼3200 compounds using approved agents in the USA, Europe, and Japan, and employed a pipeline amenable to HTS in monolayer cultures. Cold Spring Harbor Laboratory designed a screening approach to maximize the discovery of compounds selectively cytotoxic to hM1 cancer cells and fibroblasts by including both the hM1 monolayer cell line and the hM1-CAF (cancer-associated fibroblast) line (SV40 immortalized). The cells were adapted to a 1536-well plate format and the primary screening against the compound library given at a single dose was performed. 23 highly-cytotoxic agents achieved more than 80% inhibition of hM1-2D growth in the screen (Table 1). Five additional drugs were specifically evaluated because they would be part of standard of care or could potentially target hM1 genetic vulnerabilities such as the amplification of the ERBB receptor tyrosine kinase family. The team selected compounds for secondary screening and full dose response assay (Figure 13), and identified a small subset of hM1-selective cytotoxic and targeted agents. The MEK inhibitor Trametinib was one of the most potent and selective agents, with an observed EC50 of 19 nM. As previously suspected, with amplification of EGFR and HER2 in hM1 cells, the pan-ErbB inhibitors, Afatinib, and Neratinib, were very effective against hM1 cancer cells. The identification of molecular targets in the ErbB and MAPK cascade is not surprising in a KRAS mutant cancer cell [10]. Disulfiram was also identified as an unexpected compound with high activity against hM1 with an observed EC50 of 316 nM. Disulfiram is currently prescribed in the USA as a treatment for chronic alcoholism due to its inhibition of acetaldehyde dehydrogenase [11]. Surprisingly, Disulfiram was also identified as a top hit in HTS assays against prostate and breast cancer cell lines [12, 13], and many anti-cancer activities in various cancer types have been described, such as proteasome inhibition [14, 15, 16, 17]. Additionally, the proteasome inhibitor Bortezomib is also a potent cytotoxic for hM1- 2D and hM1-CAF, and does not provide selective effect on the cancer cells.

**Table 1:** 23 top hits that show >80% inhibition and 5 additional compounds of interest

	hM1-CAF			hM1-2D		
Drug Name	Final EC50 (M)	Averaged Max % Response	StdDev Max % Response	Final EC50 (M)	Averaged Max % Response	StdDev Max % Response
Dasatinib	58.9E-9	80	0.58	9.4E-9	83.75	0.35
Mithramycin	157.6E-9	93	2.56	174.1E-9	89.32	0.56
Teniposide	74.7E-9	94	0.50	482.6E-9	89.59	1.74
Methylrosanilinium	499.3E-9	101	0.06	489.1E-9	93.04	0.98
Gemcitabine	1.7E-9	96	9.21E-3	760.6E-12	93.78	0.29
Dactinomycin	3.8E-9	99	0.67	1.7E-9	94.30	0.29
Aclarubicin	610.8E-9	96	0.30	475.1E-9	94.77	0.76
Emetine	182.3E-9	100	0.18	118.3E-9	94.99	0.33
Epirubicin	84.5E-9	97	0.51	179.1E-9	95.41	0.84
Daunorubicin	38.8E-9	98	0.31	100.4E-9	95.59	0.60
Homoharringtonine	17.1E-9	99	0.20	14.1E-9	96.16	0.31
Topotecan	32.6E-9	97	0.08	312.8E-9	96.72	0.81
Idarubicin	10.7E-9	97	0.34	72.2E-9	96.85	0.51
Doxorubicin	90.6E-9	100	0.15	181.6E-9	96.89	1.07
Disulfiram	184.8E-9	59	12.08	316.6E-9	97.93	1.01
Dipyrithione magnesium sulfate	459.6E-9	93	6.61	428.8E-9	98.97	0.18
Bortezomib	1.4E-9	99	0.13	2.3E-9	99.25	0.14
Carfilzomib	2.6E-9	101	0.13	4.E-9	99.39	0.06
Romidepsin	952.8E-12	100	0.11	701.E-12	99.55	0.10
Auranofin	684.E-9	99	0.21	400.9E-9	99.55	0.07
Phanquinone	310.3E-9	99	1.14	105.8E-9	99.68	0.22
Thiram	596.4E-9	82	7.34	386.8E-9	100.10	0.07
Tyrothricin	407.2E-9	101	9.98E-3	491.5E-9	100.92	0.09
Afatinib	2.8E-6	94	3.05	226.3E-9	78.23	2.26
Neratinib	5.E-6	34	6.04	516.3E-9	67.78	1.49
Trametinib	5.E-6	38	5.35	2.7E-9	70.89	0.65
Oxaliplatin	5.E-6	11	2.82	5.E-6	10.49	2.86
Fluorouracil	5.E-6	33	9.34	5.E-6	45.58	2.18

**Figure 14:**
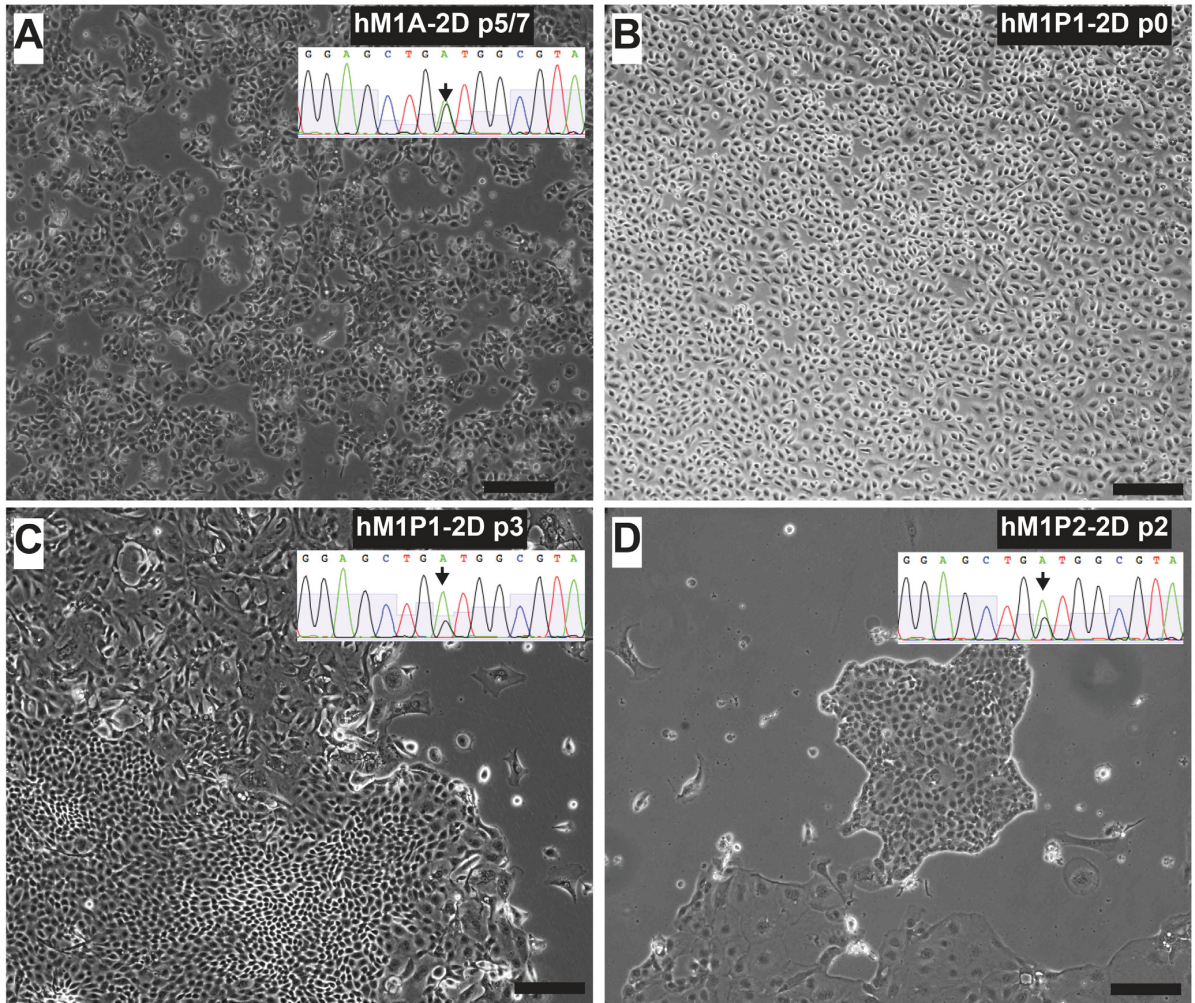
Thoracentesis-derived cell lines hM1P1 and hM1P2 contain KRAS-G12D genetic lesion Lung metastasis-derived hM1A-2D **A.** is show next to thoracentesis-derived hM1P1-2D and hM1P2-2D cell lines **C.** & **D.** All three cell lines contain the KRAS-G12D mutation (targeted sequencing of KRAS shown in inset). When received from the clinic, the pleural effusion contained a majority of mesothelial cells **B.** (Scale bar = 200 µm).

**Figure 15:**
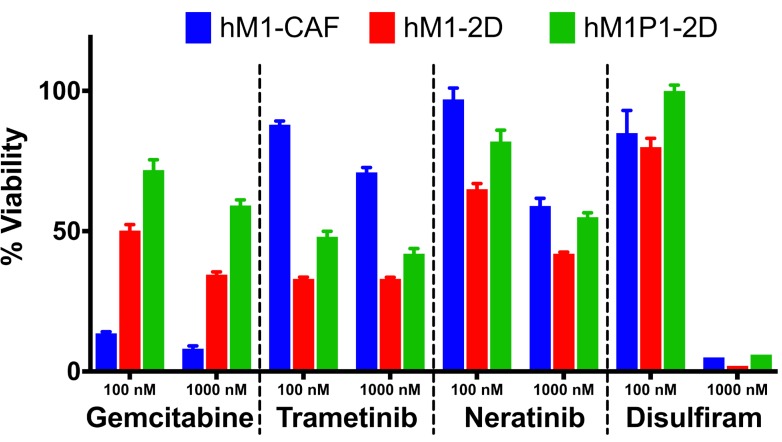
Therapeutic testing of hM1 monolayer cells Cytotoxicity of various compounds at 2 different concentrations on hM1 monolayer cell lines.

Throughout the clinical care of the patient, repeated biopsies allowed generation of additional organoid and cell line cultures. Two independent thoracenteses were performed at the Washington University Hospital in St. Louis and at MD Anderson in Houston (early 2015). The Cold Spring Harbor Laboratory team received a pleural effusion sample from each medical institution and generated two hM1P (pleural) organoid and monolayer cell lines (Figure 14). They observed that Trametinib, Neratinib, and Disulfiram maintained their activity in the newly generated hM1P cell lines, whereas the chemotherapeutic gemcitabine was now less active, hinting at an acquired resistance to ongoing therapy (Figure 15).

**Figure 16:**
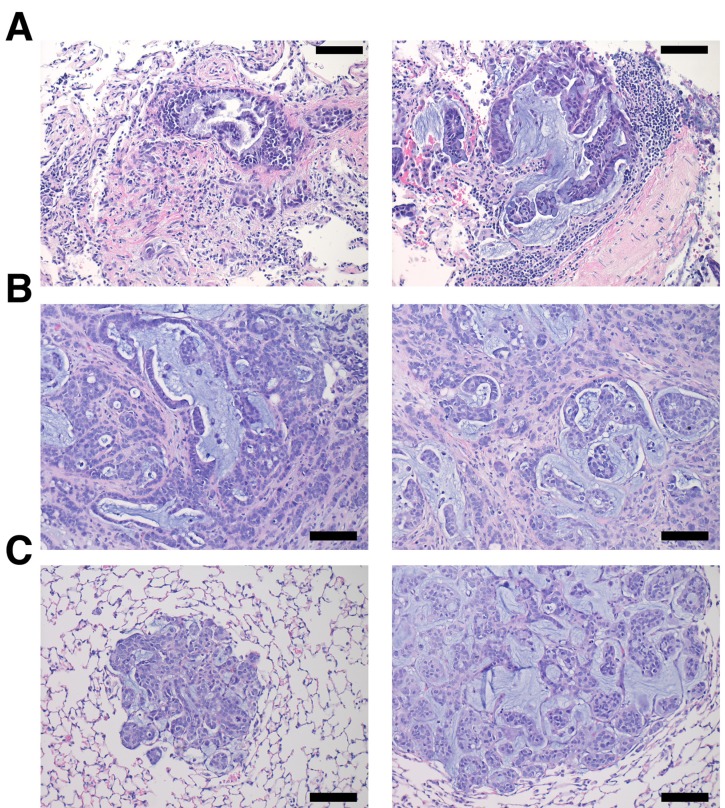
hM1 orthotopically grafted organoid (OGO) recapitulate human tumor histopathology Two representative H&E images of the patient’s lung metastatic tissue **A.** compared to large desmoplastic and mucinous mouse pancreatic tumors **B.**, and mouse lung metastases **C.** (Scale bar = 100 µm).

**Figure 17:**
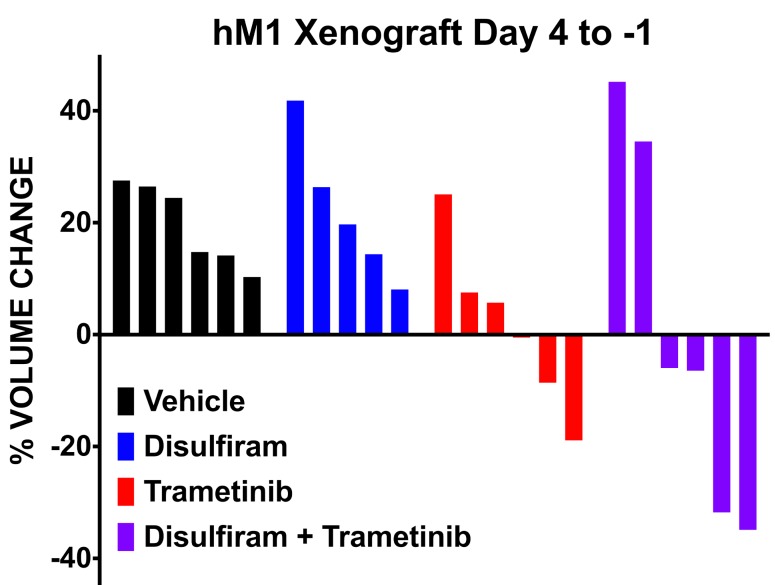
hM1 OGO mouse therapeutic study of selected compounds Response to therapy quantified by ultrasound of the tumor before and after treatment in Immuno-compromised mice (NSG) orthotopically grafted with hM1 organoid tumor pieces.

To validate targeted therapeutic strategies, the CSHL team generated hM1A mouse xenografts. The genetic alterations present in hM1 organoids, such as the KRAS-G12D and TP53-R175H mutations, render hM1A cells very aggressive when transplanted orthotopically into the pancreas of immunocompromised mice (Figure 16). Large desmoplastic tumors grew in the pancreas of the mice, and a high frequency of lung metastasis with remarkable recapitulation of the histopathology of the primary metastatic tissue was observed (Figure 16). The acquired resistance to chemotherapy seen in hM1P cells highlighted the need for targeted therapeutic options for the patient, therefore, a subset of regimens in the hM1A orthotopic xenografts was tested. The regimens tested included Disulfiram, Trametinib, and the combination of both agents (Figure 17). Whereas single agent Disulfiram did not induce a tumor regression, the MEK inhibitor Trametinib did lead to tumor regression in 2/6 mice. The combination of both agents led to the largest effect on tumor burden, with more substantial tumor regressions observed in 4/6 mice, indicating that potentially these compounds could have been beneficial to the patient. Yet, some mice did not respond to the combination (2 out of 6), highlighting the possible impact of oligoclonality on therapeutic response.

**Figure 18:**
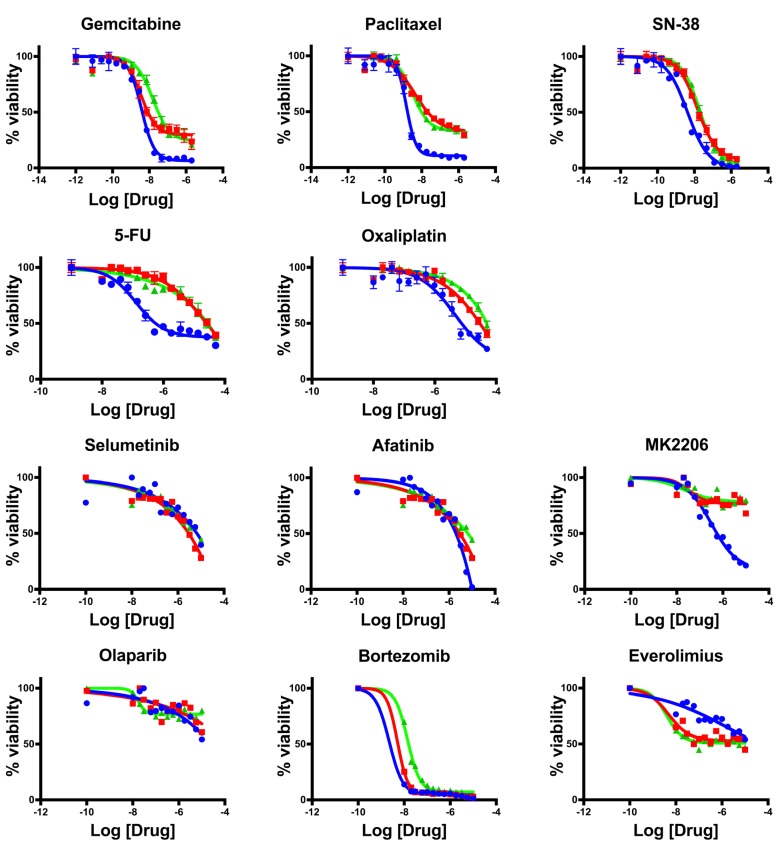
Therapeutic testing of hM1 organoid cultures Dose response assay with hM1A (blue lines), hM1E (red lines), and hM1F (green lines) organoids, for chemotherapeutic agents and targeted agents.

After the patient was hospitalized with highly progressive disease in early 2016, a core needle biopsy obtained from the Washington University Hospital in St Louis was used to generate a novel organoid line hM1E. Shortly thereafter, the patient passed away with a large tumor burden. Tissue was received from the rapid autopsy, from which was generated one final organoid line (hM1F). All of these cancer models maintained the original KRAS-G12D mutation (data not shown). To understand the drug resistance and sensitivity changes over time in serial organoid cultures, the activity of standard of care chemotherapeutic compounds and targeted agents in the hM1 organoid series was determined (Figure 18). Although the chemotherapeutic cytotoxic agents display activity in the early organoid hM1A, they lose their potency in the late hM1E biopsy and the autopsy-derived hM1F organoids. Furthermore, targeted agents such as Bortezomib and Afatinib are also less effective in the late-disease derived organoids. Some compounds do not display a change in activity, such as Selumetinib and Olaparib. The response observed in the organoids mimics the resistance observed in the patient with advanced disease, indicating that the organoid system may represent a robust system for precision medicine. Intriguingly, the testing results using the mTOR inhibitor Everolimus demonstrate increased sensitivity in hM1E and hM1F. This is in line with the pathological findings that the pancreatic ductal adenocarcinoma phenotype switched into a poorly differentiated small cell-like, neuroendocrine state. Pancreatic neuroendocrine carcinomas are known to be responsive to mTOR inhibition [18, 19], suggesting that MTOR inhibition might have been a viable clinical strategy.

### RNA and drug profiles

The Rinn lab at Harvard performed a molecular profile to understand how the tumor, hM1, responded to the top drugs screened by the Tuveson group at Cold Spring Harbor Laboratory. Specifically, the Rinn lab focused on Trametinib and Disulfiram, the former being administered to the patient, and the latter showing important synergistic effects in tumor reducing capabilities in the screen. The goal was to profile the entire transcriptome for gene-regulatory changes of mRNA, and the possible roles of long noncoding RNAs as an unexplored area. hM1 PDX models treated with Trametinib and Disulfiram (at a dosage of 3mg/kg, and 80mg/kg respectively) were analyzed in parallel with a vehicle control. The organoid treatment was performed by the Tuveson lab in quadruplicate (triplicate for Trametinib). After 7 days of exposure, tumors were harvested and snap frozen. The Rinn lab then isolated total RNA and prepared sequencing libraries (TruSeq, Illumina) for massively parallel RNA sequencing. The samples were sequenced on a HiSeq with an average depth of 39.8 million reads per replicate (437 million reads total; 50bp reads, paired-end sequencing). Finally, differential expression analyses (methods) were performed to determine genes that were significantly up or down regulated in Trametinib and Disulfiram relative to vehicle control.

**Figure 19:**
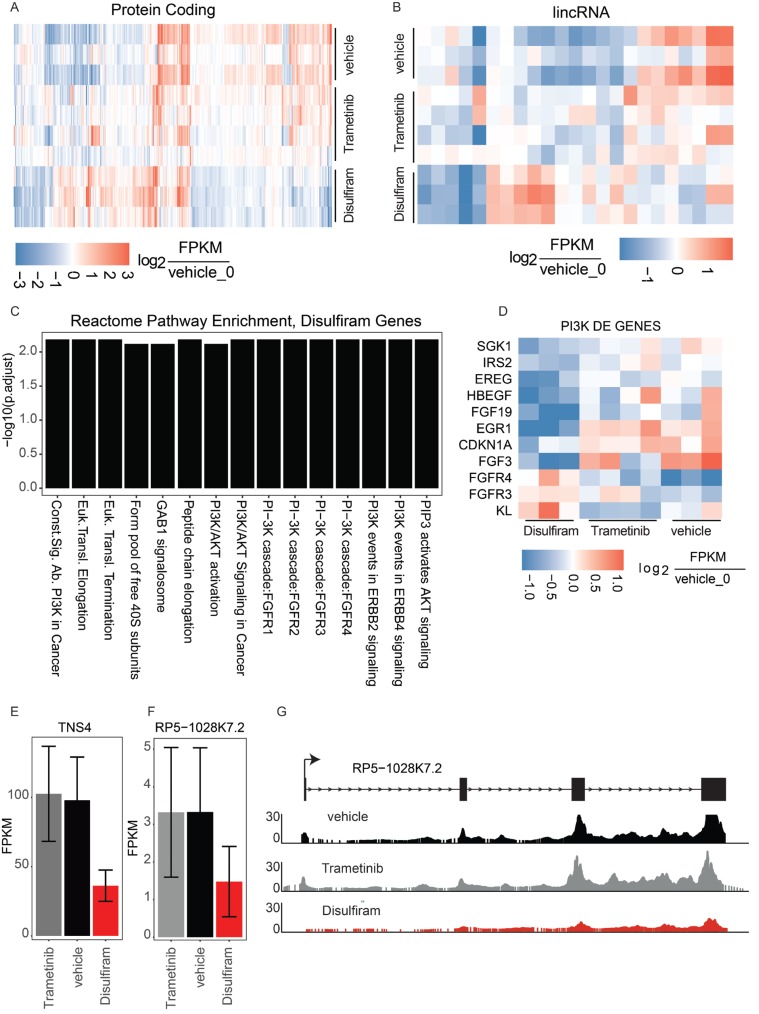
Transcriptional analysis of top drug targets **A.** Significantly dysregulated mRNA genes in either Disulfiram-vehicle or Trametinib-vehicle. Log2 fold change of each replicate is shown relative to vehicle replicate 0. **B.** Significantly dysregulated lincRNA genes. Log2 fold change of each replicate is shown relative to vehicle replicate 0. **C.** Reactome gene ontology enrichment (BH adjusted p-value < 0.01) for genes significantly dysregulated in disulfiram samples. **D.** Log2 fold change of PI3K pathway genes in each sample relative to vehicle replicate 0. **E.** Expression in FPKMs of TNS4. **F.** Expression in FPKMs of lincRNA RP5-1028k7.2. **G.** Raw RNAseq read pileup across the RP5-1028k7.2 locus, with gene structure shown above.

The largest impact was observed on transcriptional regulation in the Disulphiram-treated samples, with a total of 396 mRNA genes differentially regulated (145 up and 251 down) in treated vs. vehicle control. In contrast to Disulfiram, in the Trametinib-treated samples, only 93 mRNA genes were misregulated (48 up and 45 down) relative to the vehicle control (Figure 19A). Interestingly, it was possible to detect differential transcription of 24 lncRNAs (21 differentially expressed in Disulfiram-treated samples and 3 in Trametinib-treated samples, Figure 19B). The Rinn lab next performed gene ontology (GO) enrichment analyses to determine if there were trends in specific gene pathways or processes that were commonly mis-regulated. They were unable to find coherent gene expression changes in the Trametinib experiments (data not shown) but found a clear signature of PI3 Kinase pathway regulation in the Disulfiram-treated samples (Figure 19C). They further investigated the specific genes defining this signature and found that the vast majority were down-regulated (Figure 19D). Together, these analyses suggest that Disulfiram may strongly down-regulate PI3K signaling in tumors.

The Rinn lab next looked for long noncoding RNA (lncRNA) regulation with a particular focus on lncRNAs that are mis-regulated in Disulfiram-treated samples. They also looked for lncRNAs neighboring mRNA genes that showed coherent down-regulation, potentially implicating the lincRNA in cis regulation of the mRNA at that locus. Such a gene expression pattern would provide a new avenue of therapeutic intervention—targeting the lncRNA to down-regulate the neighboring mRNA. Indeed, they found the lncRNA RP5-1028K7.2 to be significantly down-regulated in Disulfiram-treated samples with a concomitant down-regulation of the neighboring mRNA TNS4, an emerging oncogene with ties to PI3k signaling through stabilization of c-MET [20, 21] (Figure 19E-19G). This regulatory perturbation was not observed in Trametinib samples. Collectively, the molecular profiling of PDX models of hM1 suggests a potential explanation for Disulfiram’s ability to reduce tumor volume — through reduction of PI3K signaling [22, 23], and potentially through modulation of the noncoding transcriptome.

Reads were aligned to the human genome (hg19) using hisat (with non-standard options -p 8 —qc-filter) and GENCODE annotation (v19, filtered for lincRNAs, processed transcripts, and protein coding genes) [24, 25]. Bams were quantified using cuffquant and differential expression analysis was performed with cuff diff using non-standard options -p 10, and a contrast sheet defining comparisons between Trametinib-vehicle, and Disulfiram-vehicle [26]. All analyses were performed using cummerbund and other packages from bioconductor [27, 28].

### Target determination and adoptive cell transfer

For antigen discovery, the Yee lab at MD Anderson expanded patient-derived tumor cells (hMIA-2d) to approximately 108 cells (10 x 10 cm confluent plates), then lysed them using Triton X-100. Cell lysates were incubated overnight at 4°C with gentle agitation with 1 ug HLA-A, B, C specific mAb W6/32 for every 10 mg protein. Protein A/G Ultralink resin beads were used to immunoprecipitate HLA molecules, which were then directly eluted along with tumor-associated peptides using 0.1N acetic acid in five consecutive 1 mL eluates. Purification of HLA was confirmed by Western Blot analysis and HLA-positive elutes were pooled and analyzed by tandem mass spectrometry (MS/MS), as described below. For discovery phase MS/MS, eluted HLA-bound peptides were injected onto a high-sensitivity HPLC system (Dionex 3000 RSLC), separated by reversed-phase chromatography in 0.1% formic acid water-acetonitrile on 1.8 micron C18 (Agilent Technologies) and analyzed on an Orbitrap Elite mass spectrometer (Thermo Scientific) using data-dependent acquisition. The Mascot algorithm was employed to search acquired MS/MS spectra against the SwissProt complete human protein database using 10 ppm parent mass tolerance, 0.8 d fragment ion tolerance, Met oxidation, no enzyme selectivity. Search results were cross-referenced with the appropriate MHC-binding specificities using the NetMHC 3.4 algorithm. Approximately 1800 peptides were detected in Discovery phase MS/MS, all corresponding to wild-type sequences matching proteins within the human proteome.

Based on the results of Discovery MS/MS, whole exome sequencing, and bioinformatics analysis considering target gene expression in normal tissues (GTex RNAseq databases), human pancreatic tumors (TCGA RNAseq database), and the patient’s own RNAseq analysis, 11 isotope- labeled high-confidence peptides of interest (8 mutated and 3 non-mutated) were synthesized and used as standards in a more sensitive targeted MS/MS analysis. In this analysis, retention-time windows for the synthetic peptide standards of interest were pre-determined by MS analysis of the synthetic peptides, then targeted methods for searching tumor-associated peptides were constructed using mass windows of 3 Da around each m/z. The targeted MS/MS experiments verified the presence of all 3 non-mutated peptides, but convincing MS evidence was not found for any of the predicted mutated peptides.

#### Generation of pancreatic antigen-specific CTL for adoptive therapy

Following identification of the 3 epitopes corresponding to pancreatic cancer-associated antigens (PCAA), clinical grade peptide and peptide-MHC tetramers were synthesized and used to generate PCAA-specific CTL. All clinical investigations were conducted according to the Declaration of Helsinki principles.The patient was enrolled on an approved protocol. The generation of antigen-specific CTL was performed according to methods established in the Yee Lab [29, 30]. Leukapheresis was performed to collect PBMCs, which were used as a source of stimulator (dendritic) cells and responder T cells. PBMCs were depleted of CD25+ T cells (Miltenyi Biotec Inc.) to eliminate regulatory T cells, and stimulated twice for seven days with autologous DC pulsed with PCAA peptide. DC stimulations were supplemented with IL-21 (30 ng/mL) on Day 1 and restimulated on Day 8 with IL-2 (10 U/ml) and IL-7 (10 ng/ml). Cultures that contained ≥ 5% tetramer+ CD8+ T cells were sorted using a clinical grade cell sorter (Miltenyi Nanosorter) and expanded twice using the Rapid Expansion Protocol [31]. The total production time was 6 weeks. Antigen-specificity was evaluated by testing against peptide-pulsed and antigen+ tumor targets. CTL were successfully expanded for one of the three peptides epitopes identified by targeted MS/MS. The purity and phenotype of the CTL product was defined immediately prior to infusion.

The PCAA-specific CTL line generated from patient peripheral blood specifically lysed PCAA-peptide pulsed targets; furthermore, robust and specific CTL killing of patient autologous tumor as well as an HLA-matched PCAA+ tumor line was observed, suggesting that the Yee lab had isolated a CTL line of relatively high affinity, that the tumor expressed pMHC for the PCAA epitope of sufficient density, and that such a line may exhibit tumor rejection potential following adoptive transfer. The peptide target that generated T-cells against for the patient was an HLA-A*0101-restricted peptide derived from a protein product of the gene VGLL1 (gene name = “Vestigial-like 1”).

#### Clinical protocol and course:

Under the protocol, the patient received CY (300mg/m^2^ iv) on Day -2 and then an infusion of 10^10^ polyclonal, IL-21 primed antigen-specific CTL/m^2^, immediately followed by low-dose s.c. IL-2 and PD1 blockade (3mg/kg every 2 weeks x 16 doses) [32]. Radiologic responses were evaluated according to the mWHO-based irRC Criteria [33]. The patient received an infusion of T cells (10^10^ cells /m^2^: total 19 billion PCAA, pancreatic cancer associated antigen-specific CTL) 30 days apart, each followed by low-dose subcutaneous (s.c.) IL-2 (250,000 U/m^2^ every 12h) and anti-PD1 every 3 weeks. The T-cell infusion was preceded by low-dose cyclophosphamide (CY) conditioning (300 mg/m^2^ x 1) and followed by a two-week course of low-dose subcutaneous IL-2. Although the entire regimen could be administered in the ambulatory setting, he was hospitalized for monitoring of potential cell-infusion-associated adverse events (AEs). No serious AEs were observed apart from expected transient (<24 hours) culture-negative fevers (≥38.3°C), associated with CTL-induced cytokine release syndrome, and lymphopenia lasting 10 days [34].

#### Persistence, clonality, phenotype, and function of monoclonal and polyclonal CTL *in vivo*

Transferred T cells were tracked by TCR sequence analysis. High-throughput T cell receptor V beta sequencing (HTTCS) was used to identify individual clonotypes within CTL products, and track them *in vivo* post-infusion in both peripheral blood, pleural effusion, and lung biopsy samples. T cell receptor V beta analysis of the T cell product, which was > 95% tetramer positive for the target antigen, revealed that 99% of the TCR sequences were restricted to 21 clonotypes, the dominant clone, representing 43% of all sequences. HTTCS tracking of peripheral blood samples revealed a cumulative frequency (representing all 21 clonotypes) of 1.7% of total T cells in the PBMC with the dominant clonotype comprising more than 1% of total T cells. One month later, the transferred antigen-specific T cells were present at very low but detectable levels (.03%) in the periphery; however they had accumulated in the pleural effusion biopsy (0.37%). No infiltration into the lung tumor tissue was detected.

Overall, these results suggest that a relevant tumor-associated antigenic epitope could be identified from the patient tumor sample, and was of sufficient immunogenicity to elicit autologous pancreatic tumor-reactive, antigen-specific T cells from patient peripheral blood. Isolation and expansion of such T cells for adoptive transfer was feasible, and the transferred T cells achieved relatively high frequency in the peripheral blood, with apparent enrichment in the pleural effusion, where they were detected at high frequency more than a month later. The lack of a significant clinical response may be attributable to a failure of T cells to infiltrate into the lung tissue and, notably, the loss of significant levels of the target antigen following adoptive transfer. Combination strategies to facilitate tumor infiltration and multivalent targeting (including antigen-spreading and targeting multiple antigens) may enhance clinical efficacy in future studies.

### The T-cell receptor DNA sequence representations of cells from the tumor, the leukapheresis, and the selected amplification of T-cells

**Figure 20:**
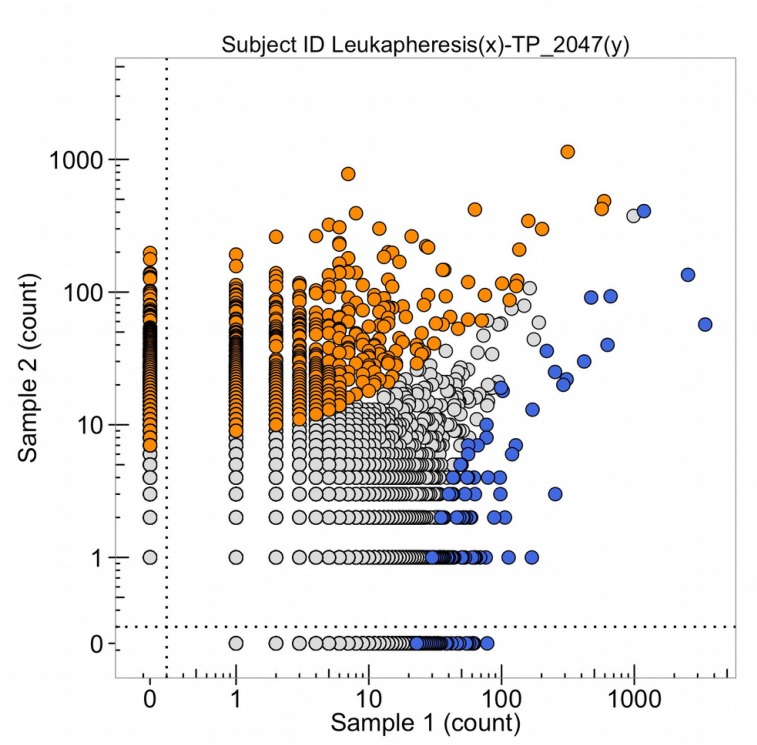
The number of DNA sequence reads of T-cell receptors from the total leukapheresis (sample 1) and from the metastatic lung tumor (TP_2047(y), obtained Nov. 2013) (sample 2) The high clonal counts of T-cell receptor sequences from the leukapheresis (in blue) and from the tumor (in orange) are presented and the sequences that are expanded clonally in both samples are in the upper right corner of the graph.

Samples of the pancreatic adenocarcinoma were taken from the metastatic tumor in the left lung extracted by thorascopic wedge resection in November 2013 for DNA sequencing of the genome, DNA sequencing of the T-cell receptor variable regions of the beta chain of the receptor (Figure 20, sample 2, blue points are each a different expanded clone sequence from the tumor) the production of organoids in cell culture, and patient derived murine xenografts. In Oct. 2015, T-cells from the patient were obtained by leukapharesis and a sample was obtained to sequence the variable regions of the beta chain of the T-cell receptor (Figure 20, orange points). The points (blue and orange) in the upper right-hand corner are clonal sequence counts (100 to 1,000 reads) in common with the tumor and the leukapharesis.

**Figure 21:**
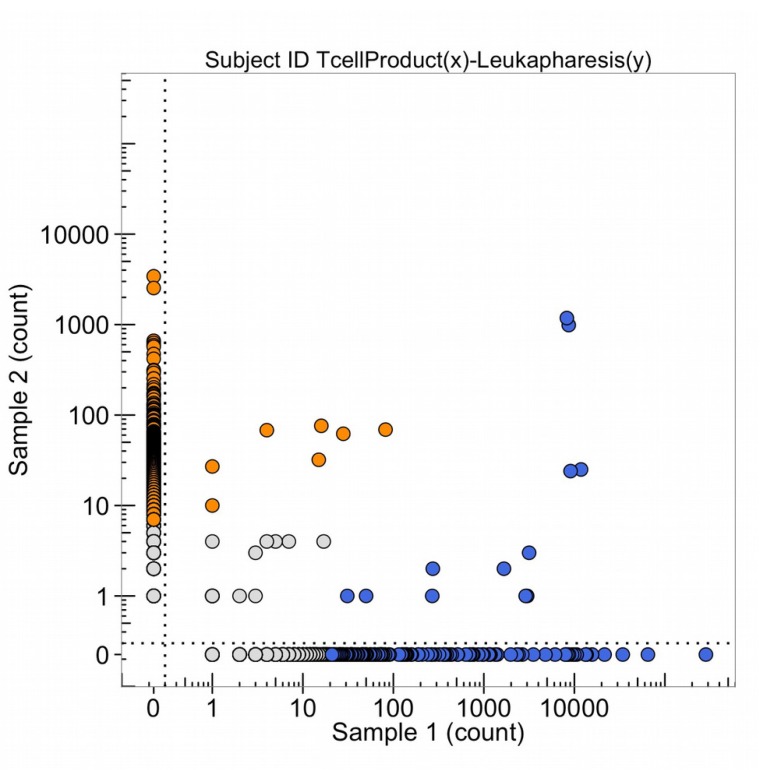
The number of DNA sequence reads of T-cell receptors from the T-cell product of the peptide amplification of the total leukapharesis (sample 1) and from the leukapheresis (sample 2) The high clonal counts of T-cell receptor sequences from the T-cell product of peptide expansion (in blue) and the leukapharesis (in orange) are presented and the sequences that are high clonal DNA sequence reads in both samples are in the upper right-hand corner.

**Figure 22:**
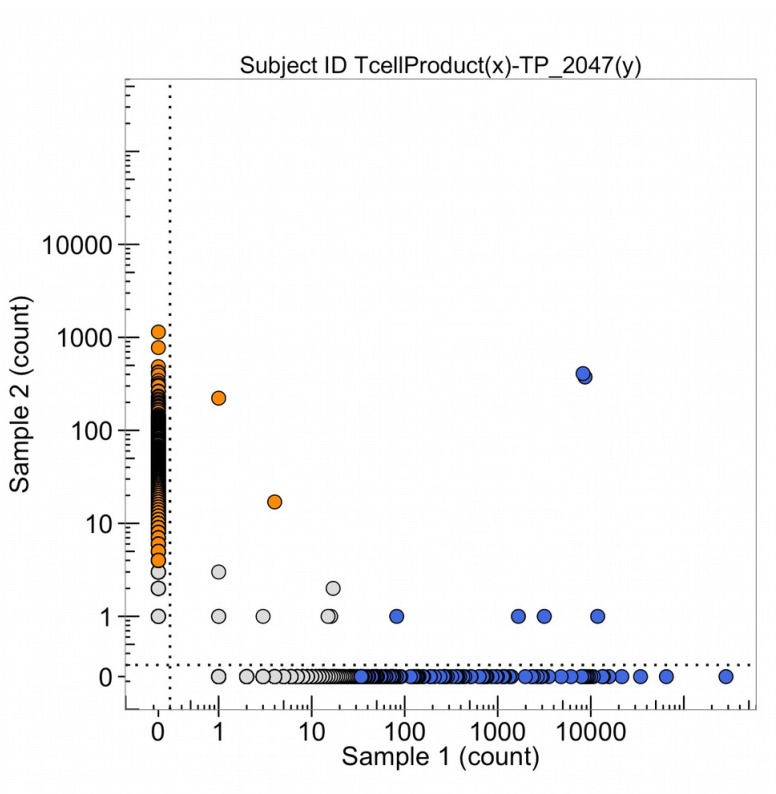
The number of DNA sequence reads of T-cell receptors from the T-cell product of the peptide amplification (sample 1) and from the metastatic lung tumor (sample 2, TP_2047(y)) The high clonal counts of T-cell receptor DNA sequence reads from the T-cell product (in blue) and the metastatic lung tumor (in orange) are presented and the two blue sequence clones in the upper right corner of the graph are in common and in high amounts in both the amplified T-cell clones (Oct, 2015) and the tumor (Nov, 2013).

Some T-cell receptor sequences are common to all individuals with the same HLA class 1 types (so-called public sequences) so these clones, with high numbers of DNA sequence reads in both samples 1 and 2 of Figure 20, do not necessarily mean detection of the same tumor antigen by an expanded DNA sequence clone in both the tumor and the blood. In October 2015, the leukapharesis sample (sample 2 of Figure 21) and the T-cell products that were expanded by a peptide (sample 1, Figure 21) were sequenced for the beta chain T-cell receptor variable region and compared. The clones in the upper right corner of the graph are found in both samples. It is notable that four blue clones (from the sample 1, T-cell product) are present in 10,000 copies, two of which are found in 10-100 copies in the leukapharesis sample 2 counts and two in 1,000-10,000 copies in sample 2 leukaphares sample. Figure 22 provides the direct comparison of T-cell clone sequences shared by the T-cell peptide expanded clones and the clones found in the original tumor two years previously (Figure 22, blue clones). Two blue clones with 10,000 sequence reads in the T-cell product have 100-1,000 reads in the tumor. This does suggest that the T-cell preparation that was infused into the patient in 2015 did contain a few clones with receptor sequences shared between the tumor and the expanded T-cell component and possibly targeting neoantigens in the tumor.

After the infusion of T-cells into the patient, (October 20, 2015) the patient continued to have tumor growth in the lung, as evidenced by increased tumor DNA markers in the blood, increased size of the lung lesions by imaging, and continued respiratory failures, although each of these observations could have been due to either an increased tumor load or a strong inflammation, killing cells in the tumor. A biopsy of the lung tumor at this time showed tissue sections with a small cell lung cancer phenotype. DNA sequencing of that biopsy material indicated that the tumor was derived from the original adenocarcinoma of the pancreas (both the RAS mutation and the p53 mutation at codon 175 were identical in both the pancreatic carcinoma and the small cell like lung cancer, as were other mutations found in the original tumor in 2013).The autopsy materials obtained in January 2016 showed that tissue sections taken from many different parts of the lung tumor had a small cell lung cancer morphology (neuroendocrine-like), whereas other sections taken from different parts of the tumor had a pancreatic adenocarcinoma-like morphology, and some samples had mixed morphologies, with both cell types. The simplest interpretation of these observations is that a portion of the tumor underwent an epithelial-mesenchyme transition (EMT) giving rise to the small cell neuroendocrine-like morphology. This epigenetic change altered the pattern of gene expression and likely altered the neoantigens expressed by the original pancreatic adenocarcinoma tumor, giving rise to a failure of the T-cell therapy that was attempted. The observation that the increased levels of the Ras DNA biomarker sequence taken from the blood prior to the October 2015 infusion of T-cells into the patient, suggested that the EMT in the lung tumor began prior to any immune-selection of the adenocarcinoma tumor cells in the lung.

Whether the immune-selection actually worked to kill the pancreatic adenocarcinoma cells, and possibly to expand the neuroendocrine-like cells is unclear. What is clear was that there was a prolonged (several years) period of chemotherapy keeping the tumor metastasis under control, and biopsies demonstrate that this gives rise to increasing clonal heterogeneity of the tumor, perhaps resulting in an EMT with epigenetic changes and altered gene expression. Both genetic and morphological heterogeneity of the lung tumor is clear from the autopsy materials. This would be expected to alter the immunological profile of possible tumor antigens. Clearly, the tumor *in vivo* evolves under therapeutic selections and there is a need for greater monitoring and speed in understanding this evolution, so that the therapeutic choices keep up with the tumor evolution and heterogeneity. This applies to both the use of immunological therapies and chemical therapies, which were tested in organoids and mice carrying this tumor. In the future, we will need accurate and rapid laboratory processing of biopsy materials so as to respond to the information collected from the tumor in real time.
